# Resting-State Brain Activity in Adult Males Who Stutter

**DOI:** 10.1371/journal.pone.0030570

**Published:** 2012-01-20

**Authors:** Yun Xuan, Chun Meng, Yanhui Yang, Chaozhe Zhu, Liang Wang, Qian Yan, Chunlan Lin, Chunshui Yu

**Affiliations:** 1 Department of Anatomy, Tianjin Medical University, Tianjin, China; 2 State Key Laboratory of Cognitive Neuroscience and Learning, Beijing Normal University, Beijing, China; 3 Department of Radiology, Xuanwu Hospital, Capital Medical University, Beijing, China; 4 Department of Radiology, Tianjin Medical University General Hospital, Tianjin, China; 5 Institute of Psychology, Chinese Academy of Sciences, Beijing, China; 6 Beijing Professor Lin Stuttering Correction Center, Beijing, China; University of Barcelona, Spain

## Abstract

Although developmental stuttering has been extensively studied with structural and task-based functional magnetic resonance imaging (fMRI), few studies have focused on resting-state brain activity in this disorder. We investigated resting-state brain activity of stuttering subjects by analyzing the amplitude of low-frequency fluctuation (ALFF), region of interest (ROI)-based functional connectivity (FC) and independent component analysis (ICA)-based FC. Forty-four adult males with developmental stuttering and 46 age-matched fluent male controls were scanned using resting-state fMRI. ALFF, ROI-based FCs and ICA-based FCs were compared between male stuttering subjects and fluent controls in a voxel-wise manner. Compared with fluent controls, stuttering subjects showed increased ALFF in left brain areas related to speech motor and auditory functions and bilateral prefrontal cortices related to cognitive control. However, stuttering subjects showed decreased ALFF in the left posterior language reception area and bilateral non-speech motor areas. ROI-based FC analysis revealed decreased FC between the posterior language area involved in the perception and decoding of sensory information and anterior brain area involved in the initiation of speech motor function, as well as increased FC within anterior or posterior speech- and language-associated areas and between the prefrontal areas and default-mode network (DMN) in stuttering subjects. ICA showed that stuttering subjects had decreased FC in the DMN and increased FC in the sensorimotor network. Our findings support the concept that stuttering subjects have deficits in multiple functional systems (motor, language, auditory and DMN) and in the connections between them.

## Introduction

Developmental stuttering is a speech fluency disorder that is characterized by repetitions, prolongations and interruptions during speech. It occurs in approximately 1% of the adult population and 5% of preschool-age children [1]. Magnetic resonance imaging (MRI) has been widely used to explore the neural substrates of stuttering. High-resolution structural MRI has shown that stuttering subjects display extensive brain abnormalities, including asymmetry [2], [3], regional anatomic variants [4], [5], and changes in gray and/or white matter densities or volumes [6], [7], [8] in brain regions involved in auditory, motor and speech processing. Diffusion tensor imaging (DTI) demonstrated that adult stuttering subjects have reduced white matter integrity in the left Rolandic operculum [9], and left ventral premotor cortex [10]. The former was also reported in stuttering children [7].

Functional neuroimaging techniques have been extensively used to explore brain activation alterations in stuttering subjects during a variety of speech and non-speech tasks. Several differences have been observed, including stuttering-induced overactivations in the motor system with right cerebral dominance, fewer left-lateralized activations in the auditory system, and selective deactivation of a frontal-temporal system implicated in speech production [11]. Stuttering subjects show less hemispheric lateralization of activation during the formulation and expression of language [12] and speech production [10], increased activation in the left anterior cingulate cortex (ACC) during silent reading and greater right hemisphere activation while reading aloud [13]. Right hemisphere involvement during language processing has also been reported in other studies on stuttering subjects [14], [15], [16] and is thought to serve a nonspecific compensatory role [17]. Stuttering subjects show reduced activation during speech perception and planning but have increased activity in the right auditory area and decreased activation in the left sensorimotor regions during speech production [18]. Although stuttering subjects show functional deficits underlying auditory processing, motor planning and execution, these differences are affected by speech manner [19]. Finally, stutter-induced activation differences are present in both overt and imagined states [20]. These techniques were also used to evaluate fluency-inducing effects on activations. Specifically, fluency-evoking tasks elicit robust activation of auditory and motor regions in the left hemisphere of stuttering subjects [21], [22]. Fluency shaping therapy also influences basal ganglia activity, which is correlated with stuttering severity [23]. A longitudinal study demonstrates that stuttering subjects show high and diffuse activation before therapy, a more distributed and left-side pattern shortly after therapy, and a right-side pattern two years later [24]. The mechanisms of fluency-inducing treatment are hypothesized to be right hemisphere mobilization and restoration of left-hemispheric lateralization of activations [11], [25], [26], [27]. Additionally, during speech, stuttering subjects show decreased functional connectivity (FC) between the left BA44 and left premotor regions and increased FC among homologous right-hemispheric structures [28], as well as abnormal effective connectivity among speech, motor and auditory areas [29], [30], [31], [32].

Several previous studies have also investigated resting-state cerebral blood flow (CBF) in stuttering subjects [12], [33], [34]. A study of 20 stuttering subjects reported global absolute blood flow reductions in stutterers as compared with fluent speakers in a resting condition [33]. However, two position emission tomography (PET) studies did not find any significant differences in resting-state CBF between stuttering and control groups [12], [34]. Given the inconsistent results, small sample sizes, and relatively old analytic methods used in those studies, our purpose was to determine whether resting-state brain activity was altered in stuttering subjects using a series of analytic methods based on resting-state fMRI data from a relatively large, homogenous sample (44 male stuttering subjects and 46 age-matched male controls). We first investigated amplitude of low-frequency fluctuation (ALFF) differences between these two groups. Then we extracted the regions with significant ALFF differences as regions of interest (ROI), and a ROI-based analysis of FC was used to test differences in FCs of these ROIs between stuttering subjects and fluent controls. Finally, we performed a data-driven analysis of FC using independent component analysis (ICA) to evaluate the differences between these two groups in the FC within several functional networks.

ALFF is considered a measure of resting-state local brain activity [35], [36], which is similar to resting-state CBF and glucose metabolic rate in PET studies. This hypothesis is supported by at least three pieces of evidence. The first is that gray matter ALFF is greater than white matter [36], which is consistent with previous research indicating that brain activity is much higher in gray matter. The second is the finding that brain regions belonging to the default-mode network (DMN) show the highest ALFF [35], [36] and glucose metabolism [37] during rest and the latter indirectly reflects the level of resting-state brain activity. Finally, ALFF in visual areas is significantly higher when the eyes open [38]. ALFF is also altered in a variety of brain diseases [35], [39], [40], [41]. ROI-based resting-state FC analysis is a popular method used to investigate time series correlations between the ROI and other voxels [42], [43] and can provide information regarding altered connections between spatially remote brain areas in diseased states [44], [45], [46]. ICA-based resting-state FC analysis is a data-driven technique that can identify several resting-state functional networks and assess FC within these networks [47], [48], [49], [50]. This method has been extensively used to explore abnormal FC in a variety of disorders [51], [52], [53].

In the present study, we hypothesized that stuttering subjects should have altered resting-state ALFF or FC because structural and functional abnormalities have been extensively reported in this disorder. It is critically important to investigate resting-state brain activity in stuttering subjects because it may lead to new understanding of the intrinsic functional differences present in stuttering subjects and can provide information regarding spontaneous neuronal activity that cannot be obtained from structural and task-based neuroimaging studies.

## Materials and Methods

### Subjects

Fifty-one adult subjects with developmental stuttering and 51 age- and sex-matched fluent controls were scanned using fMRI. Two stuttering subjects were excluded from further analysis due to excessive head motion during fMRI scanning. Given the low number of female subjects (5 cases for each group), we restricted our analyses to the 44 male stuttering subjects and 46 male controls to improve subject homogeneity. There was no significant difference in age (*P*>0.05) between the two groups. All stuttering subjects reported that they had stuttered since childhood and had not received any treatments within the past year. All subjects were right-handed [54] native Chinese speakers without histories of other language, motor, neurological or psychiatric problems. None of the control subjects had a history of stuttering. The subjects with developmental stuttering ranged in severity from 11 to 39, as assessed with the Stuttering Severity Instrument-3 (SSI-3) [55] by a speech therapist specializing in stuttering and a researcher who had received one month of training in stuttering severity assessment. The sample videos were taped while stutterers engaged in conversation, monologue, and reading tasks in front of a small audience of strangers. Later, these sample videos were independently assessed by two raters. We used interclass correlation coefficient (ICC) to test the inter-rater reproducibility and found that the ICC was high enough (94.2%) to ensure the severity assessment reliability; therefore, we reported the stuttering severity scores obtained from the speech therapist specializing in stuttering. The demographic and clinical data of all subjects are shown in Table 1. All subjects signed an informed consent form approved by the Medical Research Ethics Committee of Tianjin Medical University.

**Table 1:** Demographic and clinical data of subjects.

	Stuttering subjects	Controls
Number of cases	44	46
Sex	Males	Males
Age (years)	25.4±4.8 (17–37)	25.2±4.1 (17–37)
Onset age (years)	6.4±2.0 (2–12)	
Duration of stuttering (years)	19.1±5.5 (9–30)	
SSI-3	25.3±6.8 (11–39)	
Frequency scores	10.1±3.8 (2–17)	
Duration scores	7.8±2.3 (4–12)	

SSI-3, Stuttering Severity Instrument, 3rd Edition.

### MR image acquisition

MR images were acquired on a 3.0 Tesla MR scanner (Magnetom Trio, Siemens, Erlangen, Germany). Foam pads were used to reduce head movements, and fitted ear plugs were used to reduce scanner noise. Resting-state fMRI scans were performed with an echo planar imaging (EPI) sequence. Scan parameters were as follows: repetition time = 2000 ms; echo time = 30 ms; flip angle = 90°, matrix = 64×64; field of view = 220×220 mm^2^; slice thickness = 3 mm; and slice gap = 1 mm. Each brain volume contained 32 axial slices, and each functional run contained 180 volumes. In order to cover the whole cerebral cortex, the cerebellum could not be entirely covered in some participants with the current scan parameters. Thus, the cerebellum was excluded from the analyses. During fMRI scanning, all subjects were instructed to keep their eyes closed, relax and move as little as possible. Sagittal three-dimensional T1-weighted images with a 1×1×1 mm resolution were acquired using a magnetization prepared rapid gradient echo (MP-RAGE) sequence (repetition time = 2000 ms; echo time = 2.6 ms; flip angle = 9°).

### ALFF analysis

All preprocessing steps were performed statistical parametric mapping (SPM5, http://www.fil.ion.ucl.ac.uk/spm). The first 10 volumes of each functional time series were discarded to allow for magnetization equilibrium. The remaining 170 images were corrected for time delays between different slices and realigned to the first volume. Head motion parameters were computed by estimating the translation in each direction and the angular rotation on each axis for each volume. According to the head motion parameters, two stuttering subjects who had more than 2 mm maximum displacement in any direction (x, y, or z) or more than 2° rotation on each axis were excluded from further analysis. Each individual's T1 structural images were first co-registered with the functional images. The coregistered T1 images were segmented into gray matter, white matter and cerebrospinal fluid, and the nonlinear transformations from native space to standard space were obtained from coregistration of the T1 images with the normalized Montreal Neurological Institute (MNI) template. Then, the functional images were transformed into standard space using the same normalization parameters of T1 images and re-sampled to 3 mm cubic voxels. The normalized functional images of each subject were intersected with the first 90 brain areas (except for cerebellum) of the AAL (automated anatomical labeling) atlas [56] to obtain brain tissue masks excluding the cerebellum. The following analyses were limited to the mask.

The ALFF was computed using REST software (downloaded from http://restfmri.net, version 1.3). Because the ALFF represents the low-frequency band, linear-trend removing and temporal band-pass filtering (0.01∼0.08 Hz) were performed on the time series of each voxel in order to reduce effects of very-low-frequency drift and high-frequency noises [42], [57]. Then, the time series of each voxel was transformed to the frequency domain using fast Fourier transform (parameters: taper percent = 0, length = shortest), and the power spectrum was obtained. The square root of the power spectrum was calculated at each frequency and averaged across 0.01–0.08 Hz for each voxel. This averaged square root was taken as the ALFF [35]. For standardization purposes, the ALFF of each voxel was divided by the global mean ALFF within the brain tissue mask. The standardized ALFF of each voxel should have a value of approximately 1, and this standardization procedure is analogous to that used in PET studies [37]. Finally, spatial smoothing was conducted on the standardized ALFF map of each subject with an isotropic Gaussian kernel of 6 mm of full-width at half-maximum.

Two-sample *t*-tests were used to test ALFF differences between 44 male stuttering subjects and 46 fluent male controls. Correction for multiple comparisons was performed using Monte Carlo simulations. A corrected threshold of *P*<0.05 was derived from a combined threshold of *P*<0.01 for each voxel and a cluster size >35 voxels (AlphaSim program in AFNI software. Parameters: single voxel *P* = 0.01, 5000 simulations, FWHM = 6 mm, with gray matter mask, http://afni.nimh.nih.gov/).

### ROI-based FC analysis

Most of the preprocessing steps were similar to those used in the ALFF analysis, including discarding the first 10 volumes, slice timing, realignment, normalization to the MNI template, resampling to 3 mm cubic voxels and mask creation. After resampling, the images were smoothed with a Gaussian kernel of 6×6×6 mm^3^ full-width at half maximum. Several sources of spurious variances including estimated motion parameters, linear drift, global average blood oxygenation level dependent (BOLD) signals, and average BOLD signals in ventricular and white matter regions were removed from the data through linear regression. Finally, temporal band-pass filtering (0.01–0.08 Hz) was performed on the time series of each voxel to reduce the effects of low-frequency drift and high-frequency noises [42], [57].

Brain regions that showed significant ALFF differences between stuttering subjects and fluent controls were selected as seed ROIs. A total of 8 ROIs were defined, and the mean time series of each ROI was extracted. A detailed description of these 8 ROIs is presented in Table 2. For each subject, correlation coefficients between the mean time series of each seed ROI and that of each voxel of the whole brain were computed and then converted to *z* values using Fisher's *r*-to-*z* transformation to improve normality.

**Table 2:** Brain areas with differences in ALFF between stuttering subjects and controls.

Brain areas	Brodmann areas	Cluster size	Coordinates in MNI	*t* values
**Stuttering subjects greater than controls**				
**ROI 1**		86	−48, −6, −18	4.50
Left middle temporal gyrus	21	58	−48, −6, −18	4.54
Left superior temporal gyrus	22	20	−54, 0, −6	3.52
**ROI 2**		87	24, 48, 18	4.30
Right superior frontal gyrus	10/46	58	24, 48, 18	4.30
Right middle frontal gyrus	10/46	18	27, 48, 18	4.24
**ROI 3**		38	21, 48, 48	4.14
Right superior frontal gyrus	9	22	21, 48, 48	4.14
Right middle frontal gyrus	9	16	33, 36, 48	3.56
**ROI 4**		39	−39, −3, 45	4.11
Left premotor cortex	6	37	−39, −3, 45	4.11
**ROI 5**		77	−15, 66, 0	4.05
Left frontal pole	10	49	−3, 66, 27	3.88
**ROI 6**		36	−45, 42, 0	3.75
Left triangular portion of inferior frontal gyrus	45	33	−45, 42, 0	3.75
**Stuttering subjects lower than controls**				
**ROI 7**		198	3, −30, 78	−4.13
Right paracentral lobule	4	45	3, −30, 78	−4.13
Left paracentral lobule	4	40	−3, −36, 78	−4.04
Right precentral gyrus	4	24	12, −27, 78	−3.41
Right supplementory motor area	6	22	3, −12, 78	−3.87
Left supplementory motor area	6	20	−9, 0, 78	−3.07
**ROI 8**		54	−39, −69, 3	−3.68
Left occipitotemporal region	19/37	36	−39, −69, 3	−3.64

Abbreviations: ALFF, amplitude of low-frequency fluctuation; MNI, Montreal Neurological Institute; ROI, region of interest.

Individuals' *z*-values were entered into a random effect one-sample *t*-test to determine brain regions that showed significant positive correlations with the seed ROIs. Corrections for multiple comparisons were performed by the family-wise error (FWE) method with *P*<0.05 and cluster size >35 voxels. Then, the individuals' *z*-values were entered into a random effect two-sample *t*-test to determine group differences in the FCs with significant correlations within each group. Multiple comparisons were corrected using the same method as in the group ALFF comparisons.

### ICA analysis

The preprocessing steps were the same as the ROI-based FC analysis including slice timing, realignment, normalization, and smoothing. ICA is a powerful data-driven approach that is able to decompose noise-related components. Therefore, detrending, bandpass filtering and regressing out covariates were not performed. Finally, fMRI data for all subjects were concatenated for the group spatial ICA analysis.

Group spatial ICA was performed using the Infomax algorithm [58] within the GIFT software (http://icatb.sourceforge.net/, version 1.3 d). A two-step principal component analysis (PCA) was used to decompose the data set into 25 components. Then, time courses and spatial maps for each subject were computed and converted into z-scores [59]. In order to obtain highly robust results, ICASSO was applied 40 times employing both bootstrapping of data and random initialization. Fifteen meaningful components were identified via visual inspection and used to investigate group differences of intrinsic brain networks between the stuttering and control groups. For each component, individual maps of all subjects regardless of group were entered into random effect one sample *t*-tests and thresholded at *P*<0.05 corrected for FWE and cluster size >35 voxels, to create a sample-specific component map. These maps were used as a mask for group analyses within the corresponding component.

The z values in the individual component maps represent the fit of a specific voxel BOLD timecourse to the group-averaged component's timecourse. Thus, group analyses test the FC strength of each voxel against the whole spatial component. For each component, random effects two-sample *t*-tests were performed to test group differences in FCs within the corresponding component mask. The method of correction for multiple comparisons was the same as the ALFF and ROI-based FC analyses.

## Results

### ALFF analysis

**Figure 1:**
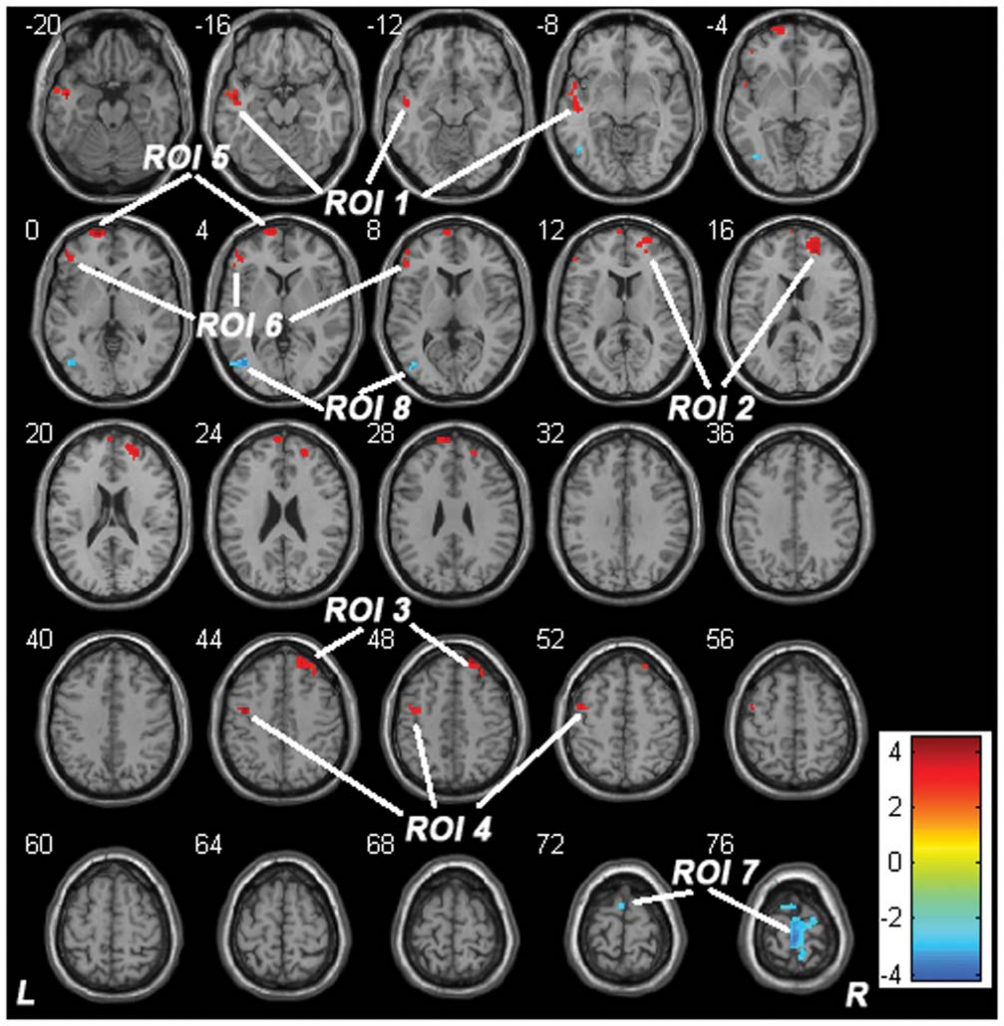
Differences in ALFF between groups. The warm color represents increased ALFF in stuttering subjects, and the blue color represents decreased ALFF in stuttering subjects. The color bars on the right side denote the *t* value. ROIs for the ROI-based functional connectivity analysis are marked in the figure. Abbreviations: ALFF, amplitude of low-frequency fluctuations; L, left; R, right; ROI, region of interest.

The ALFF was compared between groups in a voxel-wise manner. The male stuttering group had significantly (*P*<0.05, corrected) higher ALFF than the male control group in the left superior (STG) and middle temporal gyri (MTG) (auditory processing areas), the triangular portion of the inferior frontal gyrus (IFG) and premotor cortex (PMC) (speech motor areas), and bilateral prefrontal cortices (PFC) (cognitive processing areas) (Figure 1 and Table 2). In contrast, the stuttering group had significantly (*P*<0.05, corrected) lower ALFF than the controls in the bilateral supplementary motor areas (SMA) and paracentral lobules (motor areas) and the left occipitotemporal region (OT) (posterior language processing area) (Figure 1 and Table 2).

### ROI-based FC analysis

**Figure 2:**
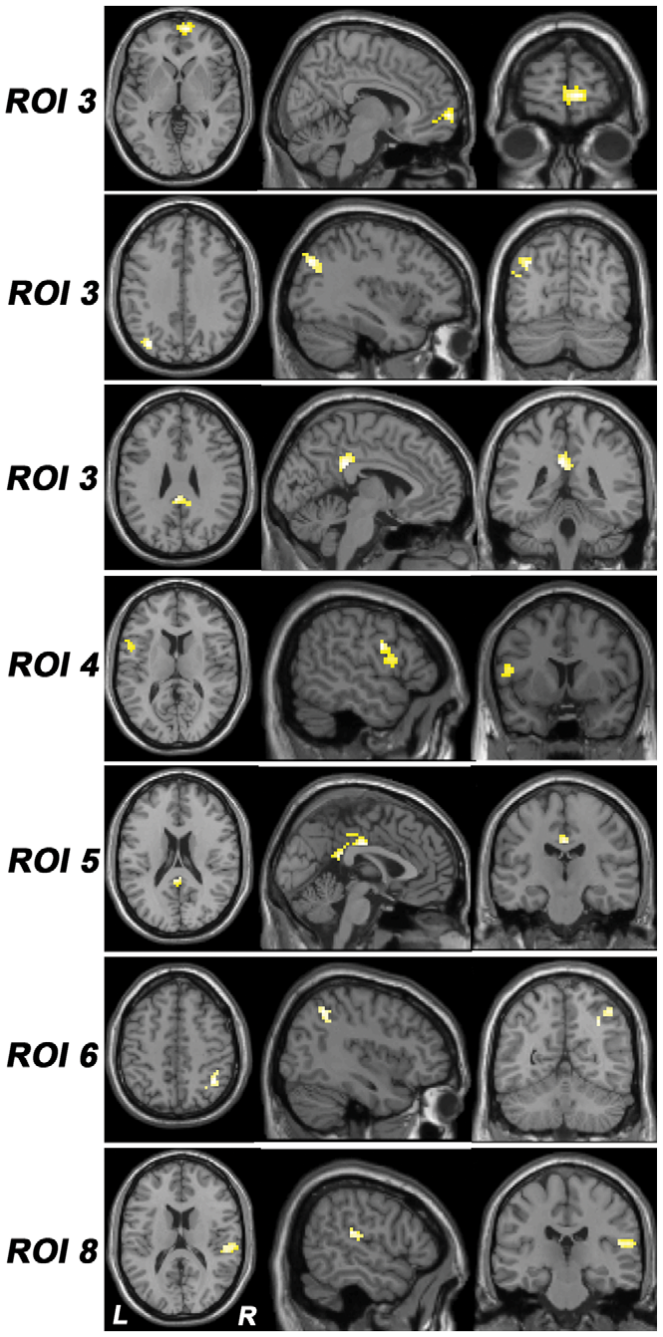
Differences in FCs between groups. This figure shows the results of the ROI-based FC analysis. The detailed information of these ROIs is shown in Figure 1 and Table 2. All of these ROIs except ROI 6 show increased (*P*<0.05, corrected) FCs in stuttering subjects. Abbreviations: FC, functional connectivity; L, left; R, right, ROI, region of interest.

Eight ROIs were selected from the ALFF analysis, and the specific locations and descriptions of these ROIs are shown in Figure 1 and Table 2. Altered FCs of these ROIs in stuttering subjects are shown in Figure 2 and Table 3. Specifically, stuttering subjects showed increased FCs between the right PFC (ROI 3) and right medial prefrontal cortex (MPFC), left occipitoparietal region and bilateral middle (MCC) and posterior cingulate cortices (PCC), left PMC (ROI 4) and left operculum of IFG (IFGop), left frontal pole (ROI 5) and bilateral PCC and MCC, and left OT (ROI 8) and right STG and inferior parietal lobule (IPL). However, stuttering subjects showed decreased FC between the left IFGop (ROI 6) and right IPL. Nevertheless, stuttering subjects did not show any significant changes in the FCs in left STG/MTG (ROI 1), right SFG/MFG (ROI 2), or bilateral SMA and paracentral lobules (ROI 7).

**Table 3:** Brain areas with differences in functional connectivity between stuttering subjects and controls.

Seed regions	Brain areas	Brodmann areas	Cluster size	Coordinates in MNI	*t* values
ROI 3 (R. PFC)	R. MPFC	10	80	9, 63, 0	4.34
ROI 3 (R. PFC)	L. OP	19/39	49	−36, −75, 36	3.67
ROI 3 (R. PFC)	B. PCC/MCC	23/31	69	−3, −39, 30	3.63
ROI 4 (L.PMC)	L. IFGop/PMC	44/6	51	−51, 0, 27	4.02
ROI 5 (L. FP)	B. PCC/MCC	23	62	0, −18, 33	3.99
ROI 6 (L. IFGop)	R. IPL	40	49	45, −45, 51	−3.20
ROI 8 (L.OT)	R. STG/IPL	40/41/42	52	57, −30, 18	3.83

Abbreviations: B, bilateral; FP, frontal pole; IFGop, operculum part of inferior frontal gyrus; IPL, inferior parietal lobule; L, left; MCC, middle cingulate cortex; MNI, Montreal Neurological Institute; MPFC, medial prefrontal cortex; OP, occipitoparietal region; OT, occipitotemporal region; PCC, posterior cingulate cortex; PFC, prefrontal cortex; PMC, premotor cortex; R, right; ROI, region of interest; STG, superior temporal gyrus.

Note: Positive *t* value represents increased functional connectivity in stuttering group, while negative *t* value denotes decreased functional connectivity.

### ICA analysis

**Figure 3:**
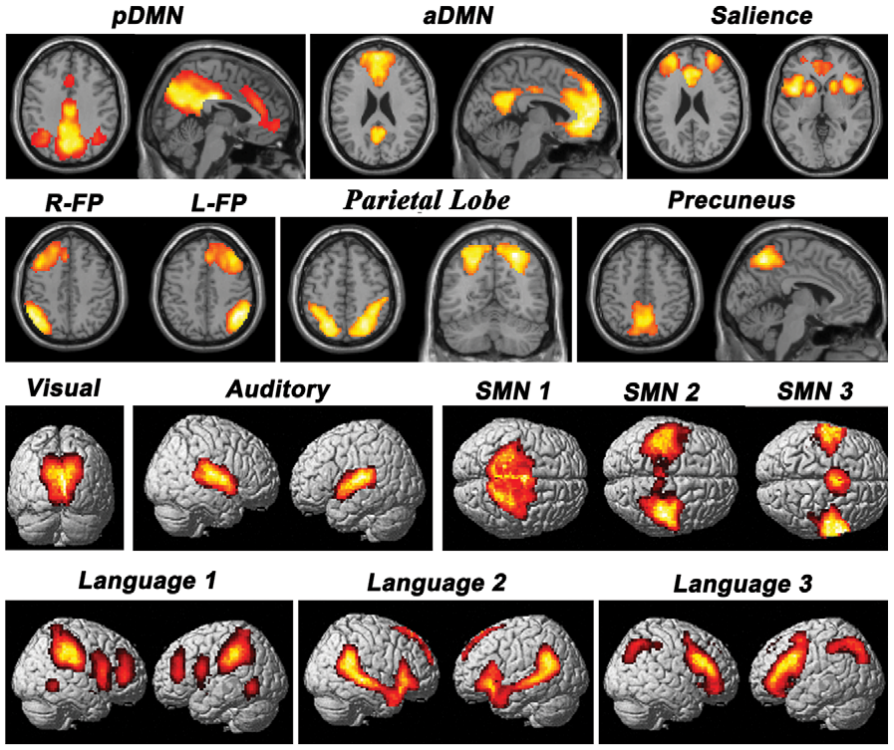
Functionally relevant resting-state networks. This figure shows the 15 functionally relevant resting-state networks resulting from the group ICA conducted on the concatenated data sets from both groups. Each component is overlaid on the structural images in standard space. Abbreviations: aDMN, anterior part of default-mode network; ICA, independent component analysis; L-FP, left frontoparietal network; pDMN, posterior part of default-mode network; R-FP, right frontoparietal network; SMN, sensorimotor network.

Twenty-five components were computed in the entire subject group by ICA. Fifteen non-noise components were selected for further analysis (Figure 3), which is consistent with previous studies [48], [49], [50]. These included the salience network, left and right frontoparietal networks, parietal lobe, precuneus lobe, visual and auditory networks, 2 components of the DMN (anterior part and posterior part), 3 components of the sensorimotor network (SMN) and 3 components of the language network (each component contains at least one of the canonical language areas, such as IFG, posterior part of the STG and MTG, and angular and supramarginal gyri).

**Figure 4:**
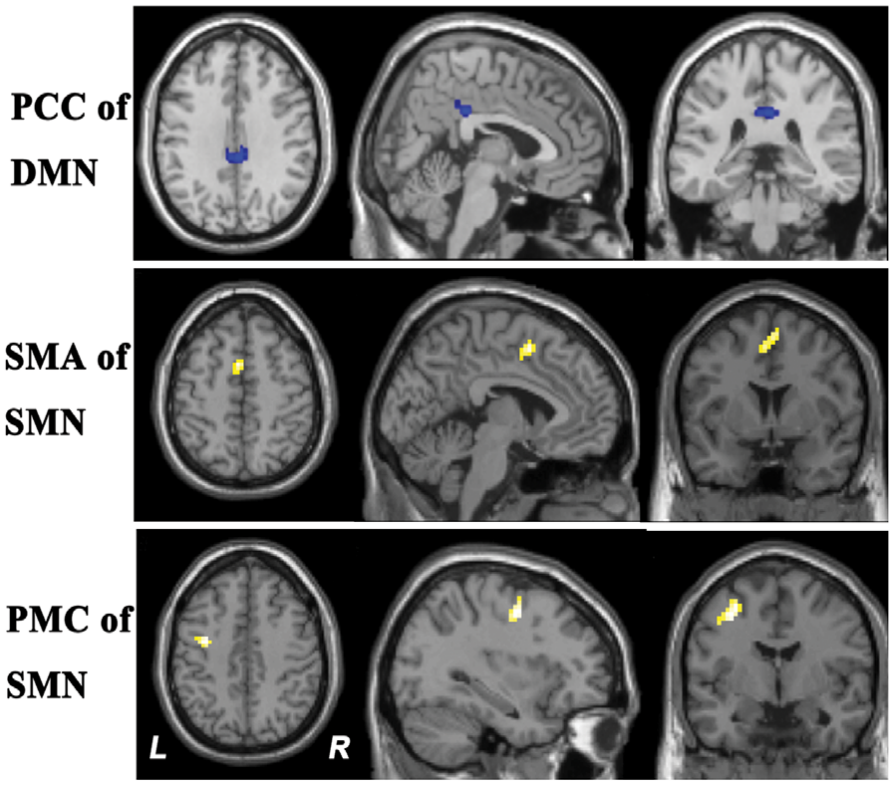
Differences in FC strength in ICA components between groups. This figure reveals that stuttering subjects showed increased FC (yellow) in the SMN and decreased FC (blue) in the DMN (*P*<0.05, corrected). Abbreviations: DMN, default-mode network; FC, functional connectivity; ICA, independent component analysis; L, left; R, right, SMN, sensorimotor network.

Voxel-wise two sample *t*-tests revealed significant group differences in the regional FC strength for components of the posterior part of DMN and SMN (Figure 4 and Table 4). Specifically, the PCC and MCC showed decreased FC with the DMN in stuttering subjects, while the left PMC and bilateral SMAs showed increased FCs with the SMN in stuttering subjects.

**Table 4:** Brain areas with differences in functional connectivity between stuttering subjects and controls as revealed by independent component analysis.

Networks	Brain areas	Brodmann areas	Cluster size	Coordinates in MNI	*t* values
DMN	B. PCC/MCC	23/31	38	6, −30, 33	−4.78
SMN	B. SMA	6	51	3, 6, 57	4.43
SMN	L.PMC	6	39	−33, −3, 48	3.79

Abbreviations: B, bilateral; DMN, default-mode network; L, left; MCC, middle cingulate cortex; MNI, Montreal Neurological Institute; PCC, posterior cingulate cortex; PMC, premotor cortex; SMN, sensorimotor network.

## Discussion

In the present study, we used a series of analytic methods based on resting-state fMRI data in a relatively large and homogenous sample to investigate the differences in resting-state brain activity between male stuttering adults and fluent controls. We found that stuttering subjects showed altered spontaneous activity in a set of brain areas involved in language, motor, auditory and cognitive processing, as well as altered FC between them. Our findings suggest that stuttering subjects have deficits in multiple functional systems and interactions between these systems, although we cannot definitively attribute these alterations to core abnormalities responsible for stuttering or lifelong attempts to deal with stuttering.

### Fluent speech production and associated brain areas

Speech production is a complex multistage process that links conceptual ideas to articulation and is controlled by cognitive components [60]. The first stage of speech production is the formation of conceptual ideas that need to be expressed, which involves several left-lateralized regions associated with semantic processing: IFG, MPFC, posterior IPL, MTG, fusiform, parahippocampal gyri, and PCC [61]. The next stage of speech production is word retrieval and sequencing to translate the combination of phonemes and syllables into a sequence of articulatory plans [60]. The left middle frontal cortex is involved in word retrieval [62], [63], the left dorsal pars opercularis is associated with sequencing linguistic and nonlinguistic events and the ventral pars opercularis is related to sequencing articulatory events [64]. Planning is followed by articulation, which is associated with initiation and coordination of movement sequences in speech articulators; this step increases activation in bilateral premotor/motor cortex, the pre-SMA, and the left putamen [65]. The final stage of speech production involves auditory and somatosensory monitoring of the spoken response, which is crucial for online correction of speech production [61] and involves the STG, posterior planum temporale, ventral SMG and cerebellum [66]. During speech production, cognitive-related prefrontal cortices are involved in suppressing competition from non-targets [61]. Theoretically, any dysfunction in speech production brain areas or in connections between them will result in dysfluency, such as stuttered speech.

### Deficits in brain areas associated with speech production in stuttering subjects

#### Brain areas associated with language perception and conceptual processing

It has been reported that post-rolandic regions, including the left posterior STG and MTG, IPL and occipital cortex are less activated during both stuttered and fluent speech in stuttering subjects [12], [15], [18]. These brain areas are involved in perceiving and decoding sensory language information and semantic processing (e.g., storing and retrieving semantic memories), which are critically important for the formation of conceptual ideas [61]. Decreased activation in these regions supports the hypothesis that stuttering speech may be caused by functional deficits in brain areas associated with language perception and conceptual processing. Our finding of decreased ALFF in the left occpitotemporal region in stuttering subjects supports this hypothesis and extends previous findings of brain activation deficits [12], [15], [18] to resting-state brain activity abnormalities.

#### Brain areas associated with phonetic encoding

The left IFG is a critical region for phonetic encoding or articulatory programming and has been shown to have reduced gray matter volume in both stuttering children [7] and adults who stutter [27]. Moreover, this reduction is positively correlated with stuttering severity, suggesting a possible origin of this disorder [27]. Normal left IFG activation during speech tasks in fluent speakers is either restricted to a small region [12] or absent [29], [31] in stuttering subjects. Unexpectedly, we found increased ALFF in this region in stuttering subjects, suggesting abnormal resting-state brain activity, although we do not know whether it is a reflection of disorder origin or compensation. Taken together, structural and functional deficits in brain areas associated with phonetic encoding may be a cause of stuttered speech.

#### Brain areas associated with articulation

The ventral primary and secondary motor cortices are important articulation regions that are functionally abnormal in stuttering subjects. When performing a nonlinguistic orolaryngeal motor task, stuttering subjects show increased activation in ventral sensorimotor and premotor areas in the left hemisphere [12]. This could represent increased effort or attention to oral motor activity; however, this may also represent fundamental differences in motor and somatosensory processing that facilitate stuttering [12]. A classical left-hemisphere activation pattern is observed in sensorimotor cortex during speech and language tasks in fluent speakers. In contrast, stuttering subjects exhibit a more diffuse bilateral activation pattern or right-hemisphere dominance [12], [16], [67]. More specifically, stuttering subjects have less activation in left sensorimotor areas during speech and non-speech perception and planning but greater activation during speech production [18]. In the present study, we found increased ALFF in the left premotor cortex and increased FC of the left premotor cortex and bilateral SMA with the sensorimotor network in stuttering subjects, suggesting functional alterations in these articulatory regions. However, we cannot specifically attribute this finding to stuttering or compensation.

#### Brain areas associated with auditory sensory feedback

Ongoing sequential and fluent speech output is dependent on auditory sensory feedback, which monitors and corrects errors online [61]. In fluent speakers, a dysfluency is detected as an “error” that is automatically corrected online. Dysfluency in stuttering subjects may be introduced by auditory perceptual defects that disrupt auditory self-monitoring, a hypothesis that is supported by a plethora of evidence. Anatomically, the left and right planum temporale (PT) show increased gray matter density [6] or volume [4] and atypical (rightward) asymmetry [3], [4] in stuttering subjects. Functionally, stuttering subjects have decreased regional cerebral blood flow (rCBF) [12], [67], reduced activation [10], [18], [29], [31], and enhanced mismatch negativity (MMN) event-related brain potential [68] in the left auditory cortex during speech tasks, suggesting a left lateralized auditory perceptual deficit that seems to underlie speech production disorder. Further support for this inference comes from studies that altered auditory feedback (delayed or frequency-shifted) reduced dysfluency in stuttering subjects by increasing auditory cortex rCBF or activation [10], [12]. Our finding of increased ALFF in the left MTG and STG suggests that abnormal auditory cortex activity can be present in the resting-state, although the functional significance of this observation requires further clarification.

#### Brain areas associated with cognitive control

We found that several prefrontal areas, including the dorsolateral prefrontal cortex (DLPFC) and frontal pole, had increased ALFF in stuttering subjects, which is consistent with previous findings that showed increased activation in these regions during language tasks in stuttering subjects[12], [27], [29]. Over-activation in these regions is inversely correlated with stuttering severity and can be abolished by fluency-shaping therapy [27]. These prefrontal areas are functionally involved in the cognitive control of complex goal-directed behavioral responses, such as motor behavior [69], [70], [71]. The DLPFC is either directly engaged during language processing, such as word retrieval [62], [63], or is indirectly involved in suppressing competition from non-targets [61]. Increased activity or activation in these prefrontal areas appears to reflect increased attention to action or increased attempts to deal with stuttering.

### Disconnection between brain areas associated with speech production in stuttering subjects

Besides regional deficits, disconnection within the speech production system is also a candidate cause for stuttering. A magnetoencephalography study of fluent speakers showed that single-word reading first activated the left inferior frontal region (articulatory preparation), followed by activation in the left ventral primary motor area (motor execution). However, order of brain activation was reversed in stuttering subjects, even when they were reading fluently [72]. This suggests that stuttering speakers have connection deficits between the left inferior frontal and left motor areas for speech production, a hypothesis that was subsequently confirmed. Both stuttering adults [9], [10] and children [7] have reduced white matter integrity immediately below the laryngeal and tongue representation in the left sensorimotor [7], [9] and premotor cortices [10], including fibers connecting the sensorimotor representation of the oropharynx with the frontal operculum and ventral premotor cortex, and fibers of the arcuate fasciculus linking the posterior superior temporal and inferior parietal cortex to frontal language areas (such as IFG). These altered anatomic connections in stuttering subjects suggest functional disconnection or imbalance between brain areas for articulatory preparation (left IFG) and motor execution (left motor cortex), as well as those for sensory perception and conceptual processing (IPL or STG). The former functional disconnection has been confirmed by two task-based fMRI analyses that found decreased functional connectivity between the left IFG and premotor cortex [28] and reduced effective connectivity between the left IFG and motor areas [29]. These findings also explain timing disturbances between left-hemisphere areas involved in language preparation and execution [72]. Unexpectedly, we found increased resting-state FC between the left IFG and left PMC in stuttering subjects, which seems contradictory with the speech task findings [28]. Although different states (rest versus task) may account for the contradictory results, we would like to reconcile these findings in a possible but largely speculative manner. Chang and colleagues [28] reported increased FC between the left IFG and left PMC in fluent controls during speech versus rest but did not find this increase in stuttering subjects. This suggests that FC between these two regions in stuttering subjects cannot increase in amplitude as large as fluent controls during speech tasks because the resting-state FC at a relatively higher level in stuttering subjects. In other words, task-state FC might depend on the level of resting-state FC, though their exact relationship remains largely unknown and requires further study. The latter functional disconnection is supported by the finding of functional imbalance between anterior forebrain regions that mediate the organization, initiation and regulation of motor activity, as well as post-rolandic regions involved in the reception and decoding of sensory information [12]. Our finding of decreased resting-state FC between the left IFG and right IPL has also been found during speech production [28], suggesting that the FC deficit between anterior and posterior language processing regions might be a candidate cause for stuttering.

### Deficits in DMN functional connectivity in stuttering subjects

The DMN is a set of brain regions that typically deactivate during cognitive tasks and includes the PCC/precuneus, ventral ACC, MPFC, and bilateral inferior parietal cortices [73]. Although a variety of functions have been ascribed to the DMN [74], [75], more and more evidence suggests that it is also involved in language processing, especially semantic (conceptual) processing. Several functional neuroimaging studies have found that most DMN brain areas engage in semantic retrieval functions during language processing [76], [77], [78], [79], [80]. Intersubject correlational analyses revealed that DMN areas are involved in narrative speech comprehension, which may be important for higher-level linguistic processes, and interface with extralinguistic cognitive, affective, and interpersonal systems [81]. Interestingly, Schafer et al. found that DMN activity positively correlated with the semantic circuit but negatively correlated with the syntactic circuit, which suggests that DMN serves as the interface between the two language sub-circuits to allow them to work together as a unified language network while functioning separately [82]. Our finding of decreased FC between the PCC/MCC and DMN suggests functional deficits in posterior brain areas associated with language perception and conceptual processing [12], [15], [18]. However, increased FC between prefrontal areas and the DMN regions may indicate increased attention to action or increased attempts to deal with stuttering.

### Non-speech motor deficits in stuttering subjects

Although stuttering is regarded as a speech motor control disorder, stuttering subjects also have deficits in non-speech motor behaviors, such as non-linguistic visuoperceptual and visuomotor deficits [83], slower movements and more errors in complex finger movements requiring timing and sequencing [84], [85], and deficits in sequence skill learning [86]. Motor function deficits may reflect more widespread difficulty with movement initiation involving the basal ganglia and SMA [84]. The basal ganglia are responsible for generating internal timing motor cues and transfering them to the SMA to support complex and sequential movements [87].The hypothesis of basal ganglia dysfunction in stuttering subjects comes from the following findings: (1) reduced rCBF in this region [88]; (2) basal ganglia activity correlates with dysfluency severity, and this activity is modified by fluency-shaping therapy [89]; (3) most subjects with acquired stuttering have basal ganglia lesions [90]; and (4) a greater incidence of involuntary movements is commonly observed with basal ganglia deficits [91]. SMA dysfunction is supported by studies that showed altered activation [12], [18] and anomalous effective connectivity [31] in stuttering subjects, by results of a case study that showed stuttering after SMA seizure [92], and by behavioral studies that showed bimanual coordination deficits [93] and difficulty in making precise movements [94], [95]. In the present study, we found that the ALFF in a cluster, including the SMA, precentral gyrus and paracentral lobule, was decreased in stuttering subjects. This cluster of brain areas is in the most dorsal part of the motor cortex and is more likely related to non-speech motor function, in contrast with the ventral motor cortex associated with speech production. Thus, this finding supports the concept that stuttering is a speech production system disorder but is also involved in non-speech motor function.

### Limitations

Several limitations of the present study should be noted. One is that we cannot exclude the influence of physiological noise because we used a relatively low sampling rate (TR = 2s) for multi-slice acquisitions. With this sampling rate, respiratory and cardiac fluctuations may be present in the time series data, which may reduce the specificity of low-frequency fluctuations [57]. Although we used band-pass filtering of 0.01–0.08 Hz to reduce this physiological noise [96], the filtering cannot completely eliminate it. Moreover, subtle changes in a subject's breathing rate or depth, which occur naturally during rest at low frequencies (<0.1 Hz), have been shown to be significantly correlated with fMRI signal changes throughout gray matter and near large vessels [97], [98]. As suggested by Birn et al. [97], we cued the subjects to breathe at a relatively constant rate and depth to partly reduce such effects. However, this procedure may explain group effects of our findings because the breathing patterns were reported to be significantly different between stutters and fluent controls [99], [100]. Because the lack of heart rate and respiration recordings are an important limitation of our study, these physiological signals should be recorded and regressed out during the resting-state fMRI analysis in future studies.

Although we found altered resting-state brain activity and FC in stuttering adults, we cannot attribute these alterations to the cause of stuttering, attempted compensation, or less optimal or optimal repairs [27]. These questions could be answered by investigating resting-state brain activity and connectivity in recovered and unrecovered stuttering children, and persisted, assisted and unassisted recovered stuttering adults; these alterations could be correlated with the severity of or improvement in dysfluency. Answers to these questions may pave the way for the use of resting-state fMRI, an easily performed technique, as a tool to monitor therapeutic effects in stuttering subjects.

Many pieces of evidence have suggested that stuttering subjects are not a homogeneous population; instead, they can be further divided into different subtypes [101], [102]. For example, based on responsiveness to amphetamine and D2-receptor blockers, stuttering subjects have been divided into “stimulant responsive” and “D2-blocker responsive” groups [103]. Furthermore, delayed auditory feedback can enhance fluency in a stuttering subgroup with atypical (rightward) PT asymmetry, but not in a subgroup with typical (leftward) PT asymmetry [3]. In the present study, we did not perform subgroup analyses because none of the proposed classification systems has received wide recognition or has been routinely applied in research or clinical spheres [101]. The existence of possible subgroups may partly account for the inability of our results to pass stricter statistical correction for multiple comparisons, such as FWE.

### Conclusions

In the present study, we found that stuttering subjects have altered resting-state brain activity in broadly distributed brain areas involved in motor, language, auditory and cognitive processing, as well as altered FC between these brain areas. Combining these results with those of other stuttering studies, we propose that stuttering subjects have deficits in multiple functional systems and inter-connections between them, although we cannot exclude the fact that some of these findings represent compensatory mechanisms. This hypothesis suggests that a combination of different therapeutic methods aimed at correcting functional deficits at different levels might be a plausible way to improve the efficacy of stuttering therapy. More importantly, if stuttering subjects can be correctly divided into biologic subgroups with different anatomic and functional changes and therapeutic responses, the efficacy of stuttering therapy and the prognosis of stuttering subjects would be largely improved.
